# Mapping digital popularity: Analyzing the network attention patterns of national forest parks based on Douyin (Tiktok) data

**DOI:** 10.1371/journal.pone.0344983

**Published:** 2026-03-27

**Authors:** Shan Zhang, Qiuchan Gu

**Affiliations:** School of International Tourism and Public Administration, Hainan University, Haikou, China; University of Ferrara, ITALY

## Abstract

The study measures the Network Attention of national forest parks based on user data from the Douyin platform, utilizing the rank-size rule and Jenks natural breaks method to analyze their spatial distribution characteristics, and employing a geographic detector to explore influencing factors. The results indicate a significant imbalance in the Network Attention distribution of national forest parks, characterized by a steep downward trend. The top parks have high attention but are few in number, while the bottom parks are numerous but receive less attention. Further investigation reveals that the spatial differentiation of Network Attention results from the synergistic drive of multiple factors. Regional economic development and the level of internet penetration emerge as critical single drivers, while factor interactions universally exhibit enhancement effects. This study provides empirical support for formulating differentiated digital dissemination strategies for national forest parks, enhancing social influence, and fostering public participation, holding significant implications for promoting ecological protection and the sustainable development of eco-tourism.

## 1. Introduction

The report of the 18th National Congress of the Communist Party of China first explicitly proposed the concepts of “promoting green development, circular development, and low-carbon development” and “building a beautiful China,” further enriching the connotation of ecological civilization construction and elevating it to the level of national strategy. These initiatives aim to promote harmonious coexistence between humans and nature. Against this backdrop, ecotourism, centered on the concept of sustainable development, has rapidly developed and received extensive attention from both the government and society [1]. Ecotourism not only focuses on environmental protection but also emphasizes the provision of cultural ecosystem services (CES). Cultural ecosystem services refer to the non-material benefits provided by ecosystems, which are related to cultural, spiritual, and aesthetic aspects. These services enhance public awareness, participation, and value perception of the natural environment, including cultural landscapes, traditional knowledge, and spiritual fulfillment, thereby promoting a harmonious relationship between humans and nature [2]. As important venues for ecotourism, national forest parks serve as key nodes linking nature conservation with human recreational activities, offering opportunities for the public to connect with nature and experience ecological civilization [3–6]. Strengthening the construction and management of national forest parks can effectively promote the development of ecotourism while contributing to the dissemination of ecological values and the protection of the ecological environment [7].

Currently, national forest parks have begun to utilize new media technologies for promotion and management [8]. Government agencies collaborate with social media platforms to release official tourism information and ecological education content, thereby enhancing public awareness of the value of forest parks and attracting more visitors [9,10]. The rise of social media has significantly transformed how information is accessed and interactions occur, profoundly impacting the promotion strategies for tourist attractions and public engagement [11]. Effectively increasing public attention to these protected areas has become a new challenge. Therefore, studying the spatial distribution of network attention and its influencing factors provides a more effective way to analyze and understand the influence and attractiveness of national forest parks among the public, identify key influencing factors, and further provide strong social support for ecological conservation.

The study of network attention has become a hot topic in the interdisciplinary fields of social sciences and information sciences in recent years, focusing on how to quantify, analyze, and interpret the attention of individuals or groups and its changes through online platforms [12,13]. In this field, user engagement is a comprehensive concept that encompasses the entire process of interaction between users and online content, from initial recognition to subsequent behavioral expressions, including behaviors such as following, viewing, liking, commenting, and sharing [14]. Among them, network attention serves as an important component of user engagement, reflecting the initial connection users establish with content through actions like following an account or becoming a fan. Deeper interactions such as liking, commenting, and sharing form a more complete picture of user engagement. There is a close relationship between these two; although follower count can be seen as an initial expression of user engagement, it does not necessarily lead to higher levels of interaction behaviors (such as likes, comments, and shares), as these behaviors may be influenced by various factors such as content quality [15]. Therefore, studying network attention not only helps to reveal the initial manifestation of user behavior but also provides valuable insights for further exploring the multiple factors that influence user interaction behaviors.

In terms of research methods, the measurement and analysis tools for network attention have undergone continuous evolution. Early on, with the widespread use of search engines such as Baidu and the accessibility of their search data, researchers began to quantify network attention using tools such as Google Trends [16], Baidu Index [17–19], and Baidu Heatmaps [20]. For instance, Li et al. used Baidu Index to obtain networke attention data for 66 of China’s first 5A-rated tourist attractions from 2006 to 2007, revealing that spatial network attention to these tourist sites was a precursor to their real visitor numbers [21]. With the launch of Sina Weibo, marking the beginning of China’s era of social media networks, users began building their personal social networks by sharing and searching for information. The rise of this new form of social media provided scholars with a new way to study network attention by analyzing shared content on platforms like Weibo [22]. For example, Yang Weishan et al. used Sina Weibo to represent the public’s attention to low-carbon cities, and found that there was a significant gap in the perceptions between the public and scholars regarding low-carbon cities [23]. In recent years, with the rapid development of short video platforms such as Douyin and the diversification of content ecosystems, the number of users has continued to grow. As of September 2023, Douyin’s monthly active users have reached 743 million, maintaining a leading position among similar platforms. In this context, metrics such as Douyin’s follower count not only reflect trends in user attention but have also become emerging indicators for studying trends in network attention. Scholars have used this data to explore various spatial patterns of network attention in tourist attractions and urban agglomerations [24,25]. Some studies have approached this from a geographical perspective, exploring the interaction mechanism between the new virtual space created by short video content and physical space [25]. Luo et al. (2023) [26] analyzed the spatial differences in network attention and the driving factors of network attention for red tourism sites in China, based on data such as the number of Douyin users and topic volume. Additionally, Wu et al. (2023) [24] analyzed the spatial differentiation patterns of network attention in the Chengdu-Chongqing urban agglomeration using the number of Douyin followers as a metric.

In conclusion, existing research has established a solid foundation for understanding the application of network attention in tourism and geography. Methodologically, the data sources for research have evolved from early search engine indices to social media content, and have further expanded to include interactive metrics from short video platforms. In terms of research scope, studies have transitioned from macro-level examinations of cities and popular tourist destinations to more specific themes, such as red tourism. However, research on the network attention of national forest parks—key entities that integrate ecological value with cultural ecosystem services—remains underdeveloped. Particularly, with the rise of short video platforms, such as Douyin, as dominant information carriers, there remains considerable potential to deepen the research based on user interaction data from these platforms. Against this backdrop, this paper investigates the spatial patterns and influencing factors of network attention for short videos related to national forest parks on the Douyin platform, uncovering the social influence characteristics of these parks in the digital age. The study aims to assist managers and planners in identifying potential opportunities and challenges in the development of ecotourism, while providing a more comprehensive and nuanced perspective for the sustainable management and promotion of high-quality tourism in national forest parks.

## 2. Data sources and research methods

### 2.1 Data sources

#### 2.1.1 List of national forest parks data.

**Fig 1:**
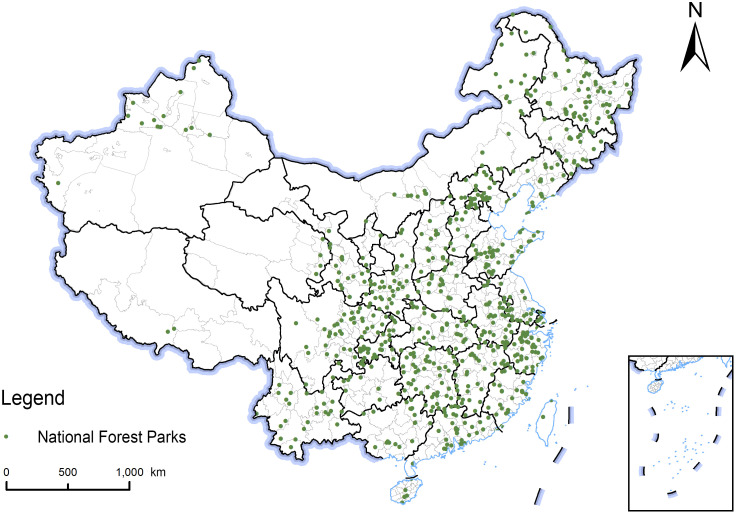
Spatial distribution of national forest parks in China. The map was created using QGIS software. The base map shapefiles were generalized from publicly available administrative boundary data, including Natural Earth (http://www.naturalearthdata.com/), which is in the public domain.

Based on the List of National Forest Parks released by the National Forestry and Grassland Administration and provincial forestry bureaus, a total of 906 national-level forest parks were identified as research objects (excluding data from Hong Kong, Macao, and Taiwan). The geographic coordinates of each park were collected using the Baidu Map Coordinate Picker System (http://aqsc.shmh.gov.cn/gis/getpoint.htm) to obtain the spatial location data of the parks (Fig 1).

#### 2.1.2 Data collection.

**Fig 2:**
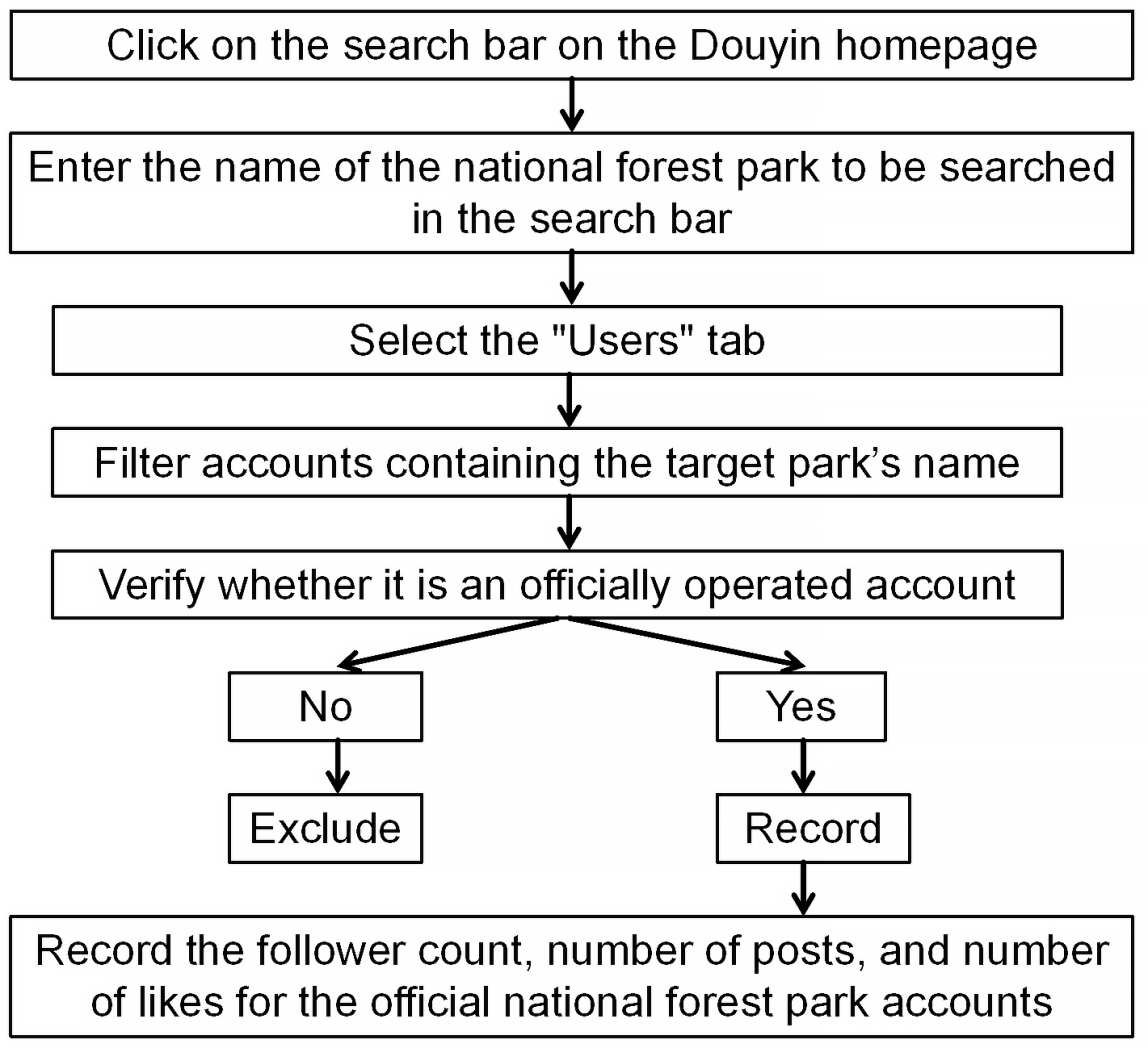
Search route of data collection.

This study systematically collected network attention data for each national forest park from the Douyin platform (https://www.douyin.com), including key indicators such as the follower count of official accounts, the total number of posts, and the number of likes. The data was extracted and organized manually, covering the period from the launch of Douyin to March 25, 2024. The data collection process is as follows: First, the exact name of the target national forest park was entered into the search bar on the official Douyin platform website. Then, users containing the park’s name were filtered from the search results, and their status as official accounts was verified. If the account was confirmed as official, the follower count, video likes, and video post count from the park’s official Douyin page were recorded; if the account was not official, it was excluded from the study (Fig 2). According to statistics, 128 national forest parks have official Douyin accounts. Furthermore, the analyses of influencing factors is based on municipal-level data, primarily sourced from official statistical yearbooks, municipal government work reports, and statistical bulletins on national economic and social development. All data collected in this study are publicly available. We confirmed that the data collection and analysis method complied with the terms and conditions for Douyin.

### 2.2 Research methods

#### 2.2.1 Rank-size rule.

The rank-size rule (also known as Zipf’s Law) is an important tool for studying the development status of cities or other systems. By analyzing the correlation between size and rank, it reveals the degree of concentration and disparity within the system [23,24]. In the field of tourism studies, this method can be used to analyze and quantify the relationship between the network attention rankings of tourist attractions and their relative sizes. It helps compare the degree of concentration and disparity in attraction scales. The core of this approach is to establish a mathematical relationship between the network attention of forest parks and their ranks, thereby further interpreting the structural differences and developmental trends within the system.The mathematical expression of this rule is:


$$lnP_{i}=lnP_{max}-qlnR$$ (1)

In this context, P represents the network attention score of the i-th forest park, P_max_ denotes the score of the forest park with the highest network attention, and R indicates the rank order of the forest parks. The parameter q, estimated through linear regression analyses and also known as the Zipf index, reflects the distribution characteristics of network attention among forest parks. Analyzing q helps to assess the internal variation within the forest park system: a q-value close to 1 indicates a relatively balanced distribution; a q-value greater than 1 suggests a pronounced primacy effect or polarization within the system; whereas a q-value less than 1 implies greater dispersion, indicating that no dominant park exists and network attention is more evenly distributed among multiple destinations.

#### 2.2.2 Jenks natural breaks classification method.

The Jenks classification method (also known as the natural breaks method) is a data grouping approach based on minimizing within-group variance and maximizing between-group variance. By identifying natural intervals in the data, this method optimally groups similar values to maximize differences between categories, resulting in a more objective classification outcome [25]. In this study, the Jenks method was used to define the levels of network attention received by national forest parks and to analyze their spatial distribution patterns.

#### 2.2.3 Geodetector.

The Geodetector is a statistical analysis tool used to detect and analyze the relationships between variables in spatial data and their correlations with geographic spatial distributions. This method is primarily employed to identify the key factors influencing the spatial distribution of a given phenomenon and to analyze the extent to which the interaction of these factors impacts spatial distribution [26]. Its expression is:


$$\text{q}=\text{1}-\frac{\text{1}}{\text{n}\sigma^{\text{2}}}\sum_{\text{i}=\text{1}}^{\text{m}}{\text{n}}_{\text{i}}\overset{\text{2}}{\underset{\text{i}}{\sigma }}$$ (2)

In the formula, q represents the explanatory power of the influencing factor on the network attention of national forest parks; $n_{i}$ and $\overset{\text{2}}{\underset{i}{\sigma }}$ denote the sample size and variance of the i-th layer, respectively. A larger q-value indicates a stronger influence of the factor on the distribution characteristics of network attention, while a smaller q-value indicates a weaker influence [27,28].

## 3. Results and analyses

### 3.1 Rank-size structural characteristics of network attention

The data for the network attention, reputation, and number of posts of national forest parks were subjected to double-logarithmic fitting with their rank order to generate rank-size plots, with $R^{\text{2}}$ values all exceeding 0.7, indicating that the data conform to the rank-size rule. The results show that the q-values for follower count, number of likes, and number of posts are all greater than 1, indicating that the network attention system of the parks is unbalanced overall.

**Fig 3:**
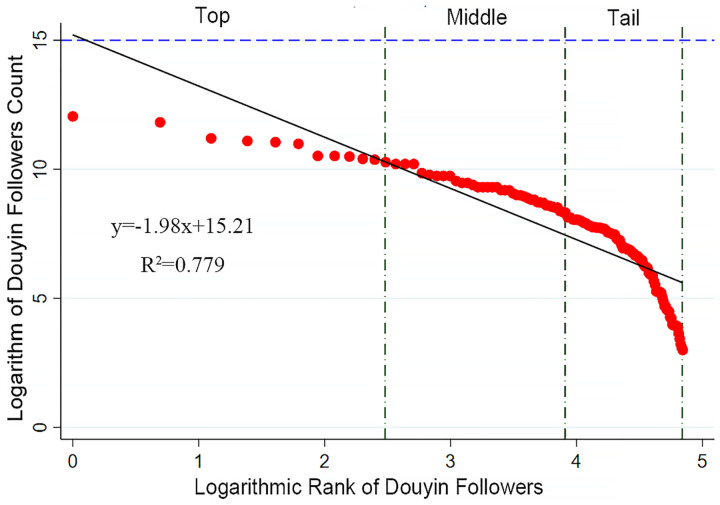
Rank-size structure of users’ network attention of Douyin-Followers Count.

Specifically, the follower count of top-ranked national forest parks demonstrates a significant advantage over other parks, forming a small, distinct leading group (Fig 3). This small group’s superiority in follower count indicates high popularity and strong appeal on the Douyin platform. The follower counts of mid-ranked parks show a relatively flat downward trend on a logarithmic scale, with relatively small differences between them, indicating a certain degree of competitive balance. Although their follower counts are lower than those of the top-ranked parks, these parks still maintain relatively high attractiveness and a stable fan base. The follower counts of lower-ranked parks show a pronounced downward curve, with those at the tail end exhibiting an almost vertical decline, reflecting weaker performance in attracting followers. Parks in this segment may face challenges such as low visibility, insufficient marketing efforts, or unfavorable geographic locations, making it difficult for them to effectively attract and retain followers.

**Fig 4:**
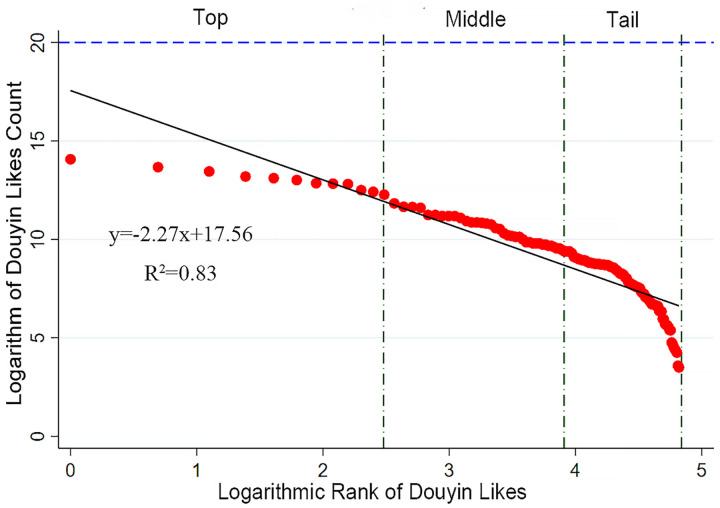
Rank-size structure of users’ network attention of Douyin-Likes Count.

In the rank-size analyses of the number of likes, a similar distribution pattern is observed (Fig 4). The top-ranked parks show a significant lead in the number of likes, reflecting outstanding performance in user engagement and content quality. The number of likes for mid-ranked parks is relatively uniform, indicating a certain level of consistency and competitiveness in maintaining user interaction. The lower-ranked parks show a densely distributed yet sharply declining trend in likes as the rank order increases, especially at the lower end of the chart, where a steep drop is observed. This suggests that the lower-ranked parks face considerable challenges in attracting likes, potentially due to lower content quality, limited visibility, or lower levels of user engagement.

**Fig 5:**
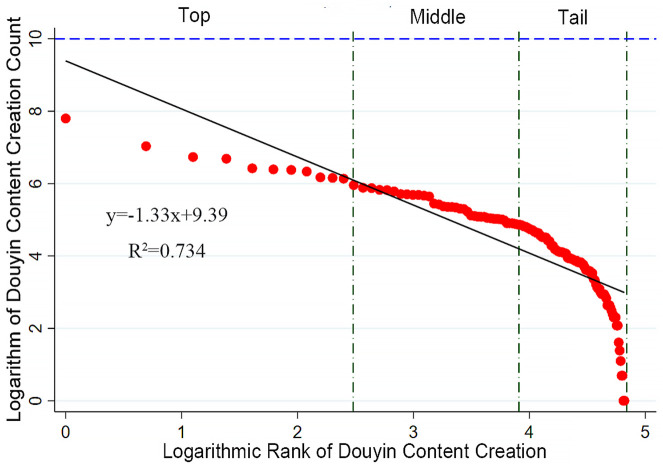
Rank-size structure of users’ network attention of Douyin-Content Creation Count.

In the rank-size analysis of the number of posts, the top-ranked parks show a highly dispersed yet small total output, indicating that only a few parks are particularly active in content creation (Fig 5). The number of posts for mid-ranked parks is relatively consistent and accounts for a significant proportion of the total, indicating a relatively balanced and competitive level of content production. The lower-ranked parks show a dense and continuously decreasing trend in the number of posts, similar to the trend in follower counts, indicating significant shortcomings in content creation among the lower-ranked parks Table 1.

**Table 1:** Structural characteristics of douyin social media data for national forest parks.

Category	High	Medium	Low
Follower Count	Absolute dominance; significant advantage held by a select few	Balanced stability; minimal inter-park variation	Distinct disadvantage; small scale with rapid decline
Engagement	Highly active; robust user engagement	Sustained stability; moderate interaction maintained	Generally low; challenges in garnering interaction
Content Production	Sparse; high activity concentrated in a few hubs	Spatially uniform and widespread	High density yet consistently sluggish

### 3.2 Spatial distribution characteristics of network attention and engagement

**Fig 6:**
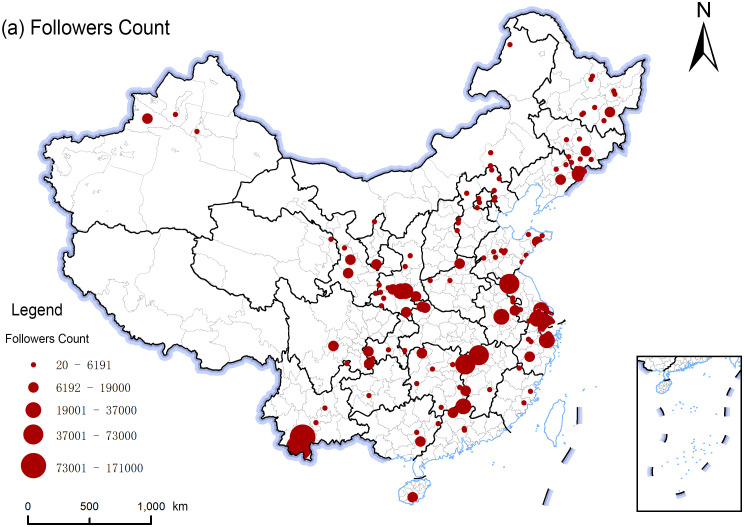
Spatial distribution of attention of national forest park network based on Followers Count. The map was created using QGIS software. The base map shapefiles were generalized from publicly available administrative boundary data, including Natural Earth (http://www.naturalearthdata.com/), which is in the public domain.

**Fig 7:**
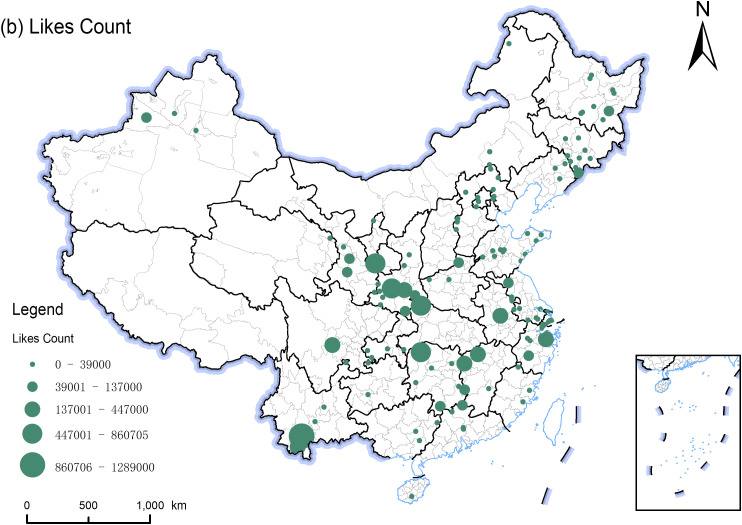
Spatial distribution of attention of national forest park network based on Likes Count. The map was created using QGIS software. The base map shapefiles were generalized from publicly available administrative boundary data, including Natural Earth (http://www.naturalearthdata.com/), which is in the public domain.

In analyzing Douyin social media data for national forest parks, the spatial distribution characteristics of network attention were revealed using the natural breaks method in ArcGIS. First, Douyin accounts with higher follower counts are primarily concentrated in the Yangtze River Delta (Jiangsu, Zhejiang, and Shanghai) region, reflecting higher Network Attention in this area (Fig 6). This distribution pattern is likely associated with the region’s higher level of socio-economic development and well-established tourism infrastructure. Additionally, follower counts in southern Shaanxi and Yunnan also exhibit a relatively large scale, forming local highlands of attention. Further analysis reveals that the spatial distribution of like counts does not entirely correspond to the geographic concentration of follower counts. In contrast to the agglomeration of follower counts in coastal regions, areas with high like counts appear more densely in inland provinces such as Shaanxi, Hunan, and Yunnan (Fig 7). This indicates that regional advantages in follower counts do not directly translate into equivalent advantages in interaction volume, reflecting a divergence between Network Attention and user engagement. A higher follower base does not necessarily lead to higher-frequency interaction behaviors. Network Attention focuses on the breadth of connections established between users and accounts, representing the public’s potential willingness to attend; whereas user engagement more intuitively reflects the actual intensity and scale of interactions triggered by content. Since they differ in formation mechanisms and core focuses, they exhibit differentiated spatial distribution patterns.

**Fig 8:**
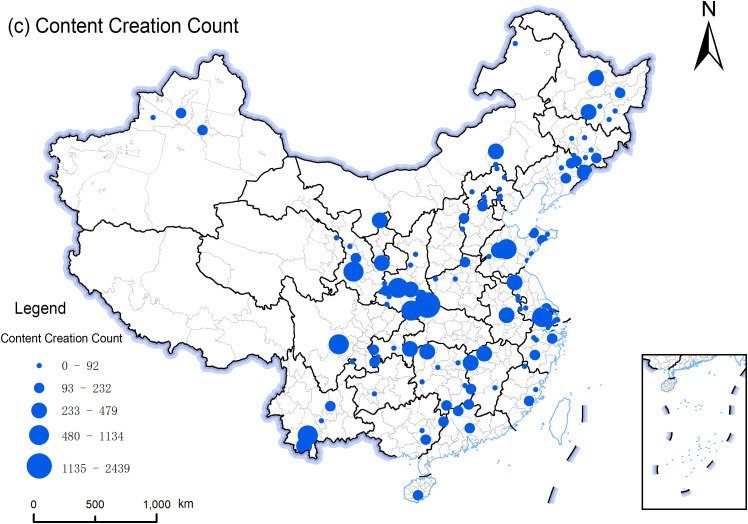
Spatial distribution of attention of national forest park network based on Content Creation Count. The map was created using QGIS software. The base map shapefiles were generalized from publicly available administrative boundary data, including Natural Earth (http://www.naturalearthdata.com/), which is in the public domain.

Compared with follower counts and likes, the distribution of content production is more uniform, with high volumes of content output across multiple regions, demonstrating the widespread activity of Douyin creators (Fig 8). Overall, these three indicators—follower counts, like counts, and content production—reflect the geographic distribution of user attention, interaction, and creation, revealing significant geographic differentiation and regional heterogeneity in user behavioral patterns regarding National Forest Park content on Douyin.

## 4. Influencing factor analyses

### 4.1 Selection of influencing factors

Existing literature on the factors influencing Network Attention indicates that it is generally shaped by the combined influence of multiple complex factors, such as the level of economic development, technological innovation capacity, and internet penetration [29–31].When specifically analyzing the influencing factors of Network Attention towards forest parks, additional dimensions such as the region’s own ecological resource endowment and population size must also be considered [32–34]. Regarding the selection of specific indicators, Regional Gross Domestic Product (GDP) is used to characterize the overall economic strength of an area, as its development level provides foundational support for tourism infrastructure construction and market-oriented operations [35]. The total number of granted patents is used as a proxy variable for regional innovation capacity, reflecting the potential driving role of technological innovation on online dissemination through new business formats such as smart tourism [36]. The number of mobile internet users is adopted to measure the coverage level of regional digital infrastructure, an indicator that influences information reach efficiency and the effectiveness of online publicity [37]. Forest coverage rate is a core metric for assessing regional ecological advantages and the natural appeal of parks [38]; areas with higher forest coverage typically possess more attractive landscapes and favorable ecological conditions [39], forming the content basis for Network Attention. The size of the regional permanent population is chosen to characterize the potential tourist market [40], providing the social foundation for word-of-mouth communication about forest parks. This study aims to investigate the formation mechanisms of the spatial patterns of Network Attention from the perspective of the macro-regional context.

### 4.2 Analyses of influencing factors

First, the Geodetector was employed to identify the key factors driving the spatial differentiation of Network Attention. As indicated in Table 2, the explanatory power (q-value) of the influencing factors regarding the spatial heterogeneity of Network Attention for national forest parks follows the order: level of economic development (X_1_)> level of internet penetration (X_3_)> technological innovation capacity (X_2_)> ecological resource endowment (X4)> population scale support (X_5_). Specifically, X_1_ and X_3_ passed the significance test, indicating that the level of regional economic development and the prevalence of network infrastructure are the core driving factors leading to regional disparities in Network Attention for national forest parks.

**Table 2:** Indicator system of influencing factors.

Indicator Category	Indicator Variable	Unit	Geodetector		Ridge Regression
			q-statistic	p-value	Standardized Coefficient
Economic Development	Regional GDP (X_1_)	10,000 CNY	0.1151	0.0033	0.012
Technological Innovation	Number of Granted Patents (X_2_)	Items	0.0817	0.1119	0.034
Internet Penetration	Mobile Internet Users (X_3_)	10,000 Households	0.1086	0.0204	0.000
Ecological Endowment	Forest Coverage Rate (X_4_)	%	0.0724	0.3073	0.012
Population Size	Regional Resident Population (X_5_)	People (in 10,000s)	0.0412	0.5782	0.031

**Fig 9:**
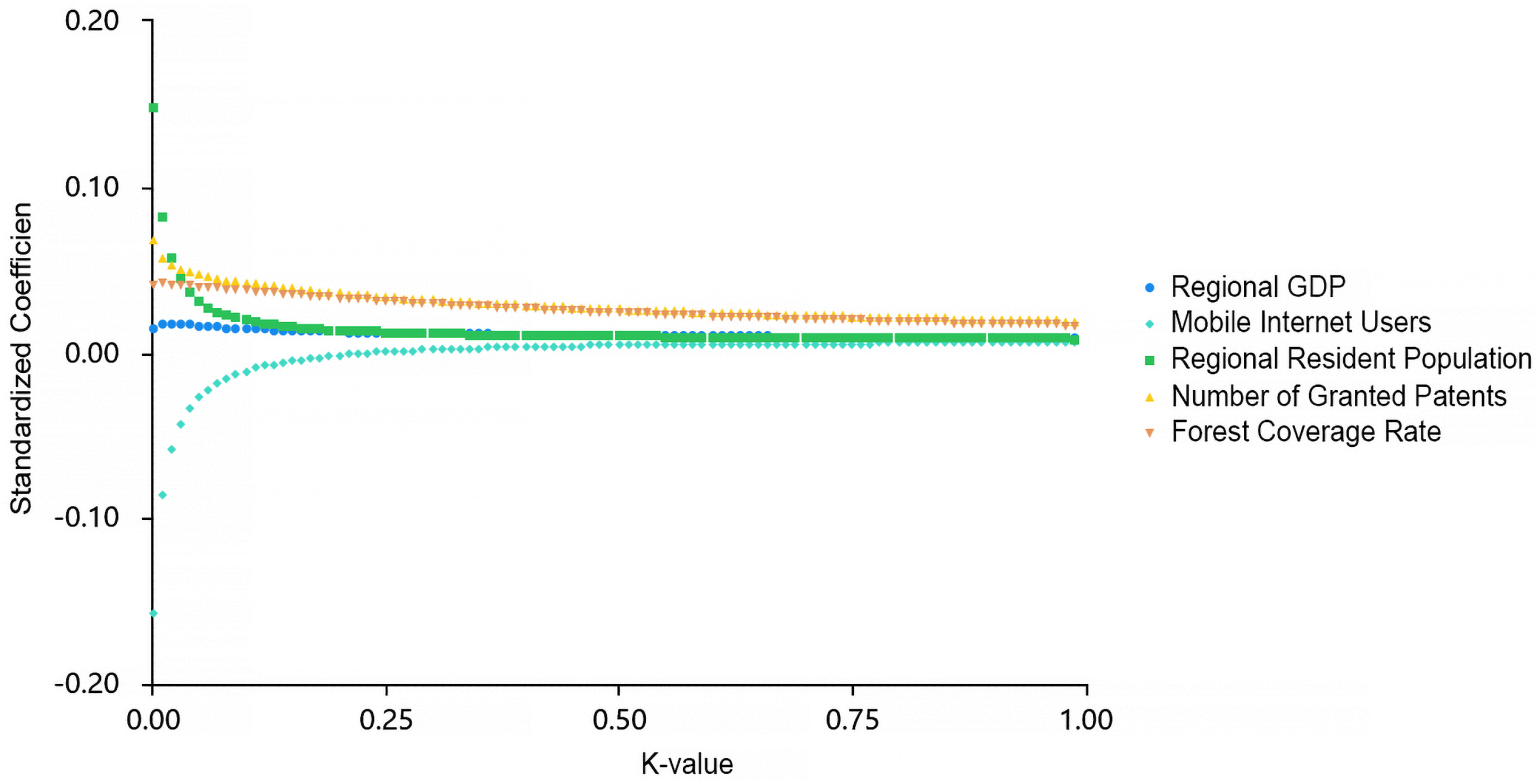
Ridge trace plot of the ridge regression for the influencing factors of Network Attention on national forest parks.

To address the severe multicollinearity among the factors (with initial VIF values far exceeding 10), this study further employed ridge regression for robust estimation. When the ridge parameter (K-value) increased to 0.25, the ridge trace plot (Fig 9) showed that the curves of all standardized coefficients stabilized, and the VIF values for all variables dropped below 10. This indicates that the multicollinearity issue was effectively controlled, thereby ensuring the robustness of the estimation results.

The results indicate that the level of regional economic development exerts a stable positive influence on Network Attention. Comparatively, regions with stronger economic capabilities typically possess more mature markets and diversified media resources, enabling them to allocate sufficient resources to support the construction and maintenance of forest parks. Furthermore, these regions can enhance public awareness of forest parks through extensive promotional campaigns. Moreover, existing studies have shown that residents in economically developed regions typically demonstrate higher willingness to consume and participate in leisure tourism and ecological experience activities [41], laying a foundation of potential online user groups for Network Attention towards national forest parks in these areas.

Secondly, the standardized coefficient for the level of internet penetration gradually stabilizes at a positive value as the K-value increases. As the infrastructure for information dissemination, the increase in internet penetration directly expands the potential audience scope for digital content. Simultaneously, widespread internet infrastructure lowers the technical and data thresholds for both media and users to create and disseminate related short videos [42]. Collectively, these factors provide structural support for the formation and diffusion of Network Attention for national forest parks from the dual dimensions of dissemination channels and content supply.

### 4.3 Interaction analyses of influencing factors

Based on the interaction detector results from the Geodetector, it was found that the q-values of all factor combinations were greater than those of any single factor. This indicates that the interactions between factors generally enhanced their individual explanatory power (see Table 2), suggesting that the network attention of National Forest Parks is the result of the synergistic action of multiple factors. Notably, the interaction explanatory power of ecological resource endowment and the level of internet penetration ranks the highest among all combinations. This suggests a complementary mechanism between digital infrastructure and ecological resources. Empowered by the mobile internet, the intangible values carried by national forest parks—such as landscape aesthetics and spiritual respite—have gained broader dissemination space, facilitating the efficient conversion of “high-quality ecological content” into “online traffic attention Table 3.”

**Table 3:** Results of influencing factors by interactive detection.

Interaction Effect	X1	X2	X3	X4	X5
**X1**	–	–	–	–	–
**X2**	0.1319	–	–	–	–
**X3**	0.0889	0.1640	–	–	–
**X4**	0.2692	0.1953	0.3737	–	–
**X5**	0.1404	0.2300	0.1344	0.3566	–

## 5. Conclusion and discussion

### 5.1 Conclusion

This study conducted an in-depth investigation into the impact of social media data on the network attention of national forest parks, focusing on the spatial distribution characteristics of key indicators such as follower count, number of likes, and number of posts related to short videos of forest parks on the Douyin platform. The study employed the rank-size rule, the Jenks natural breaks method, and the Geodetector to comprehensively analyze the effects of various social and economic factors on network attention. The main findings of this study are as follows:

(1) According to the rank-size rule, the network attention system of national forest parks as a whole is imbalanced. There are relatively few top-ranked national forest parks, which are scattered, while mid-ranked parks exhibit a more even distribution. In contrast, lower-ranked parks account for a larger proportion and show significant disparities, indicating a need to improve the overall online visibility of forest parks.
(2) Regarding spatial distribution, Network Attention and user engagement exhibit distinct characteristics of geographic differentiation. Douyin accounts with high follower counts are primarily concentrated in the Yangtze River Delta (Jiangsu, Zhejiang, and Shanghai), whereas regions with high like counts are predominantly found in inland areas such as Shaanxi and Yunnan. This spatial non-synergy indicates a divergence between Network Attention and user engagement; a substantial follower base does not necessarily translate into higher-frequency interaction behaviors. In contrast, content production across parks is relatively balanced, with high volumes of content output observed in multiple regions.
(3) In terms of influencing factors, the spatial differentiation of Network Attention for national forest parks is the result of the synergistic action of multiple factors. Among these, the level of regional economic development and the level of internet penetration serve as critical single driving factors. Furthermore, interaction effects between factors are universally present and generate enhancement effects. Notably, the interaction between ecological resource endowment and the level of internet penetration exhibits the strongest explanatory power, highlighting the significant role played by the “ecological-digital” interaction effect in driving Network Attention.

### 5.2 Discussion

As an important indicator of the digital influence of national forest parks, network attention directly reflects public awareness and interest in these parks. Enhancing the network attention of national forest parks can provide stronger social support for ecological conservation. However, the network attention of these parks remains unevenly distributed, with a large proportion of lower-ranked parks showing significant disparities. Therefore, efforts should focus on strengthening the following two aspects:

(1) The study reveals that the Network Attention system of national forest parks exhibits a distribution pattern characterized by a “prominent head and a long tail.” This structural feature indicates that the Cultural Ecosystem Services (CES) values—such as aesthetic appreciation, recreation, and education—inherent in the majority of forest parks have not yet been fully translated and effectively disseminated in the digital sphere. Integrating the research findings, while regional economic development and internet penetration are critical external factors influencing attention, their enhancement often entails long-term structural adjustments. Therefore, for “tail” parks, the key to elevating Network Attention lies not in replicating the resource-intensive content models of “head” parks, but in shifting towards differentiated dissemination paths that are lightweight, localized, and highly accessible. Specifically, strategies should encourage thematic creative campaigns rooted in local characteristics, such as “Capturing the Four Seasons of the Most Beautiful Forest” or “Storytelling of My Hometown Forest Park.” Characterized by low entry barriers, strong emotional bonds, and rapid mobilization, such activities can effectively stimulate the local public’s willingness to create and share. This approach helps tail parks gradually accumulate a repository of localized digital content with a sense of identity and viral potential, thereby laying a foundation for the sustainable elevation of their Network Attention.
(2) The spatial non-synergy between Network Attention and user engagement indicates that a high volume of followers does not necessarily translate into high levels of interaction. Consequently, for national forest parks that have already accumulated a certain degree of attention, the pivotal challenge lies in converting their existing attention advantages into deep, incremental user engagement. Specifically, these parks should reduce their reliance on homogeneous landscape displays and instead shift towards interactive designs centered on their core Cultural Ecosystem Services (CES) values. For instance, leveraging their unique ecological resources (e.g., specific rare species or iconic landscapes), parks can initiate lightweight interactive activities such as “Ecological Q&A,” “Seasonal Change Check-ins,” or “Best Viewpoint Voting” within short video content or on their account homepages. Such designs aim to transform unidirectional content dissemination into bidirectional invitations for participation, thereby catalyzing a shift in follower identity from “viewers” to “participants” and even “co-creators.” Ultimately, this enhances both the frequency and quality of deep interaction behaviors such as likes, comments, and shares.

This study, based on social media data from Douyin, analyzed the network attention characteristics of national forest parks, contributing to the promotion of ecotourism development in China. However, there are some limitations. For example, the data is concentrated on a single platform, which may not fully capture the behavior patterns of all social media users. Additionally, while the analysis covers multiple dimensions of influencing factors, it does not fully explore how these factors influence individual and group behavior within complex social networks.Future research could be expanded in several directions. First, the scope of the study could be extended to include data from more social media platforms to obtain a more comprehensive view of network attention. Second, more advanced data analysis methods, such as machine learning and artificial intelligence, could be employed to more accurately predict and explain the dynamic changes in network attention. Lastly, qualitative research methods, such as in-depth interviews and focus groups with park visitors, could be conducted to gain deeper insights into how users interact with national forest parks through social media. These approaches would provide a more comprehensive understanding of the potential role of social media in modern environmental management and ecotourism, offering valuable insights for policymaking and practical applications in related fields.

## Supporting information

S1 Dataset The minimal data set underlying the findings of this study. This file includes the list of National Forest Parks, their geolocation coordinates, Douyin interaction metrics (followers, likes, content creation counts), and the regional influencing factor data used for analysis. (XLSX)

## References

[1] Fan G, Liu C. Visualization analysis of eco-tourism resources development research in china based on citespace. Asia Soc Sci Acad. 2021;5(3):97–107. doi: 10.51600/jass.2021.5.3.97

[2] Romanazzi GR, Koto R, De Boni A, Ottomano Palmisano G, Cioffi M, Roma R. Cultural ecosystem services: A review of methods and tools for economic evaluation. Environmental and Sustainability Indicators. 2023;20:100304. doi: 10.1016/j.indic.2023.100304

[3] Kim JS, Lee TJ, Hyun SS. Estimating the economic value of urban forest parks: Focusing on restorative experiences and environmental concerns. Journal of Destination Marketing & Management. 2021;20:100603. doi: 10.1016/j.jdmm.2021.100603

[4] Kozłowska-Adamczak M, Essing-Jelonkiewicz P, Jezierska-Thöle A. Leveraging information and communication technologies in forest ecotourism: a case study from Poland. Sustainability. 2023;16(1):56. doi: 10.3390/su16010056

[5] Cheng Y, Zhao B, Peng S, Li K, Yin Y, Zhang J. Effects of cultural landscape service features in national forest parks on visitors’ sentiments: A nationwide social media-based analysis in China. Ecosystem Services. 2024;67:101614. doi: 10.1016/j.ecoser.2024.101614

[6] Zhang H, Yu J, Dong X, Zhai X, Shen J. Rethinking cultural ecosystem services in urban forest parks: An analysis of citizens’ physical activities based on social media data. Forests. 2024;15(9):1633. doi: 10.3390/f15091633

[7] Chen Z, Fu W, Konijnendijk van den Bosch CC, Pan H, Huang S, Zhu Z. National forest parks in China: origin, evolution, and sustainable development. Forests. 2019;10(4):323. doi: 10.3390/f10040323

[8] Andreopoulou Z, Koliouska C, Lemonakis C, Zopounidis C. National forest parks development through internet technologies for economic perspectives. Oper Res Int J. 2014;15(3):395–421. doi: 10.1007/s12351-014-0147-8

[9] Criado JI, Sandoval-Almazan R, Gil-Garcia JR. Government innovation through social media. Government Information Quarterly. 2013;30(4):319–26. doi: 10.1016/j.giq.2013.10.003

[10] Pratiwi S, Juerges N. Digital advocacy at the science-policy interface: Resolving land-use conflicts in conservation forests. Land Use Policy. 2022;121:106310. doi: 10.1016/j.landusepol.2022.106310

[11] Shang Y, Mehmood K, Iftikhar Y, Aziz A, Tao X, Shi L. Energizing intention to visit rural destinations: how social media disposition and social media use boost tourism through information publicity. Front Psychol. 2021;12:782461. doi: 10.3389/fpsyg.2021.782461 34887820 PMC8650607

[12] Ripberger JT. Capturing Curiosity: using internet search trends to measure public attentiveness. Policy Studies Journal. 2011;39(2):239–59. doi: 10.1111/j.1541-0072.2011.00406.x

[13] Yang X, Pan B, Evans JA, Lv B. Forecasting Chinese tourist volume with search engine data. Tourism Management. 2015;46:386–97. doi: 10.1016/j.tourman.2014.07.019

[14] Dolan R, Conduit J, Fahy J. Social media engagement: A construct of positively and negatively valenced engagement behaviours. 2015. p. 96–117.

[15] Voorveld HAM, van Noort G, Muntinga DG, Bronner F. Engagement with social media and social media advertising: the differentiating role of platform type. Journal of Advertising. 2018;47(1):38–54. doi: 10.1080/00913367.2017.1405754

[16] Nghiem LTP, Papworth SK, Lim FKS, Carrasco LR. Analysis of the capacity of google trends to measure interest in conservation topics and the role of online news. PLoS One. 2016;11(3):e0152802. doi: 10.1371/journal.pone.0152802 27028399 PMC4814066

[17] Wang H, Wang W, Meng Y, Zhang Y. Degree of user attention to a webpage based on Baidu Index: an alternative to page view. Journal of Experimental & Theoretical Artificial Intelligence. 2013;26(2):235–49. doi: 10.1080/0952813x.2013.815281

[18] Liu Y, Liao W. Spatial Characteristics of the tourism flows in China: A Study Based on the Baidu Index. IJGI. 2021;10(6):378. doi: 10.3390/ijgi10060378

[19] Zhang G, Yuan H. Spatio-Temporal evolution characteristics and spatial differences in urban tourism network attention in china: based on the baidu index. Sustainability. 2022;14(20):13252. doi: 10.3390/su142013252

[20] Lyu F, Zhang L. Using multi-source big data to understand the factors affecting urban park use in Wuhan. Urban Forestry & Urban Greening. 2019;43:126367. doi: 10.1016/j.ufug.2019.126367

[21] Li S, Q Rong-Xu Q, Ling C. Cyberspace attention of tourist attractions based on Baidu index: Temporal distribution and precursor effect. Geography and Geo-Information Science. 2008.

[22] Cui H, Kertész J. Attention dynamics on the Chinese social media Sina Weibo during the COVID-19 pandemic. EPJ Data Sci. 2021;10(1):8. doi: 10.1140/epjds/s13688-021-00263-0 33552838 PMC7856455

[23] Yang W, Cai B, Wang J, Cao L, Li D. Study on low-carbon cities’ popularity in China. China Population, Resources and Environment. 2017;27(2):22–57.

[24] Wu X, Guan W, Zhang H, Li W. Spatial differentiation and influencing factors of online attention in Chengdu-Chongqing urban agglomeration: Based on the number of Douyin fans. World Regional Studies. 2023;32(11):130.

[25] Yawen X, Xiaoqing L, Kaichun Z, Binggeng X. Spatial pattern and influencing factors of urban tourism Douyin attention: A case study of the urban agglomeration in the middle reaches of the Yangtze River. Economic Geography. 2023;43(2):220–8. doi: 10.15957/j.cnki.jjdl.2023.02.023

[26] Luo W, Wang F, Ding Z. Spatial difference and influencing factors of Douyin’s network attention of red scenic spots in China. Economic Geography. 2023;43(3):198–210.

[27] Zipf GK. Human Behavior and the Principle of Least Effort. 1948.

[28] Chen Y. The evolution of Zipf’s law indicative of city development. Physica A: Statistical Mechanics and its Applications. 2016;443:555–67. doi: 10.1016/j.physa.2015.09.083

[29] Jenks GF. The data model concept in statistical mapping. 1967.

[30] Wang J, Xu C. Geodetector: Principle and prospective. 2017;44:411–34. 10.11821/dlxb201701010

[31] Shu R, Xiao J, Su Z. Spatiotemporal trends and factors influencing online attention for China’s tea industry. Front Environ Sci. 2023;11. doi: 10.3389/fenvs.2023.1206705

[32] Zhang L, Zhou X. Exploring the spatiotemporal structure and driving mechanism of digital village construction in China based on social network analysis and Geodetector. PLoS One. 2024;19(11):e0310846. doi: 10.1371/journal.pone.0310846 39546543 PMC11567553

[33] Zhang L, Du H, Zhao Y, Wu R, Zhang X. Urban networks among Chinese cities along “the Belt and Road”: A case of web search activity in cyberspace. PLoS One. 2017;12(12):e0188868. doi: 10.1371/journal.pone.0188868 29200421 PMC5714330

[34] Nan G, Xin-cheng Z, Lin-yan W. Spatio-temporal characteristics and influencing factors of Chinese red tourism network attention. Journal of Natural Resources. 2020;35(5):1068. doi: 10.31497/zrzyxb.20200505

[35] Shen P, Yin P, Niu B. Assessing the combined effects of transportation infrastructure on regional tourism development in China Using a Spatial Econometric Model (GWPR). Land. 2023;12(1):216. doi: 10.3390/land12010216

[36] Torres P, Godinho P. Does tourism matter to national innovation capability?. Tourism Planning & Development. 2022;20:1–26. doi: 10.1080/21568316.2022.2146740

[37] Chen M, Dong D, Ji F, Tai Y, Li N, Huang R, et al. A Study on Spatiotemporal Evolution and Influencing Factors of Chinese National Park Network Attention. Land. 2024;13(6):826. doi: 10.3390/land13060826

[38] Fan C, Zhou L, Gai Z, Shen S, Liu C, Li S. Multi-dimensional evaluation framework for the sustainable development of forest health bases and site selection for application in China. Forests. 2022;13(5):799. doi: 10.3390/f13050799

[39] Song H, Qiu RTR, Park J. A review of research on tourism demand forecasting: Launching the Annals of Tourism Research Curated Collection on tourism demand forecasting. Annals of Tourism Research. 2019;75:338–62. doi: 10.1016/j.annals.2018.12.001

[40] Wang K-L, Sun T-T, Xu R-Y, Miao Z, Cheng Y-H. How does internet development promote urban green innovation efficiency? Evidence from China. Technological Forecasting and Social Change. 2022;184:122017. doi: 10.1016/j.techfore.2022.122017

[41] Ren Y, Lu L, Zhang H, Chen H, Zhu D. Residents’ willingness to pay for ecosystem services and its influencing factors: A study of the Xin’an River basin. Journal of Cleaner Production. 2020;268:122301. doi: 10.1016/j.jclepro.2020.122301

[42] Du X, Liechty T, Santos CA, Park J. ‘I want to record and share my wonderful journey’: Chinese Millennials’ production and sharing of short-form travel videos on TikTok or Douyin. Current Issues in Tourism. 2020;25(21):3412–24. doi: 10.1080/13683500.2020.1810212

